# High-resolution adaptive imaging with a single photodiode

**DOI:** 10.1038/srep14300

**Published:** 2015-09-18

**Authors:** F. Soldevila, E. Salvador-Balaguer, P. Clemente, E. Tajahuerce, J. Lancis

**Affiliations:** 1 GROC.UJI, Departament de Física, Universitat Jaume I, E12071 Castelló, Spain; 2 Institute of New Imaging Technologies (INIT), Universitat Jaume I, E12071 Castelló, Spain; 3 Servei Central d’Instrumentació Científica (SCIC), Universitat Jaume I, E12071 Castelló, Spain

## Abstract

During the past few years, the emergence of spatial light modulators operating at the tens of kHz has enabled new imaging modalities based on single-pixel photodetectors. The nature of single-pixel imaging enforces a reciprocal relationship between frame rate and image size. Compressive imaging methods allow images to be reconstructed from a number of projections that is only a fraction of the number of pixels. In microscopy, single-pixel imaging is capable of producing images with a moderate size of 128 × 128 pixels at frame rates under one Hz. Recently, there has been considerable interest in the development of advanced techniques for high-resolution real-time operation in applications such as biological microscopy. Here, we introduce an adaptive compressive technique based on wavelet trees within this framework. In our adaptive approach, the resolution of the projecting patterns remains deliberately small, which is crucial to avoid the demanding memory requirements of compressive sensing algorithms. At pattern projection rates of 22.7 kHz, our technique would enable to obtain 128 × 128 pixel images at frame rates around 3 Hz. In our experiments, we have demonstrated a cost-effective solution employing a commercial projection display.

In the latter years, single-pixel imaging (SPI) has been established as a suitable tool in life sciences. One of the main characteristics of the technique is that it uses very simple sensors (bucket detectors such as photodiodes or photomultiplier tubes) and mathematical algorithms to recover an image^1^. This reduction in complexity on the sensing device gives these systems the capability to work efficiently in conditions where light is scarce^2^. Furthermore, single-pixel cameras have been demonstrated to obtain images at shallow depth overcoming the scattering problem^3^,^4^,^5^. There are also several approaches that exploit the simplicity of the detectors in order to acquire multidimensional information, such as 3D, polarization and spectral images^6^,^7^,^8^,^9^,^10^. However, this complexity reduction in the sensor entails an increase of computational time to recover an image when compared to conventional techniques. In the visible region of the spectrum, where pixelated sensors have acquired very high performances with low costs, this may not seem like a good trade-off. Nevertheless, in other regions of the spectra, such as infrared and terahertz, where pixelated sensors do not have such good specifications, this technique can provide huge benefits^11^,^12^,^13^,^14^.

To recover an image, SPI needs to overlap a set of masks onto the scene under study and recover the total intensity of light transmitted or reflected by the scene. The size of this set depends on the desired resolution of the image. Even for low resolution images of 64 × 64 pixels, this requires a huge amount of projections (64^2^). In spite of the fact that fast spatial light modulators (digital micromirror devices, or DMD) are usually used in these single-pixel camera architectures, this limits the speed of the acquisition process.

In order to solve this problem, compressive sensing (CS) techniques provide a method to recover the images with a number of measurements lower than the total number of pixels. This is possible because natural images tend to be sparse (i.e. only a small fraction of these projections have relevant information) in some basis of functions^15^. Despite lowering the total number of projections, the reconstruction algorithms require high computational power, which also limits the technique to low resolution images if the user wants high speed acquisition and high-speed display in real-time. Some other techniques tackle this problem by using temporal sparsity^16^,^17^.

Recently, a technique has been reported operating at sub-Hz rates with image sizes up to 128 × 128 pixels^18^. The key element of the technique is to project only the functions of the basis that have most of the information about the scene and recover the picture without using CS algorithms, thus speeding-up the display stage. Even though this approach solves the problem of the reconstruction time of the CS algorithms, to project only the important masks one must know beforehand which ones have the relevant information about the scene. Moreover, if the scene changes quickly, the a priori information of the relevant functions is rapidly lost. In order to achieve real time frame rates, it would be preferable to have a technique that does not use a priori information about the scene maintaining the benefits discussed above.

Here we propose an adaptive method for SPI that recovers images with a number of measurements lower than the number of pixels of the scene, with finer details than CS techniques (given the same number of measurements) and lower reconstruction times. This approach does not need to have a priori knowledge of the object and only collects information about the relevant parts of the scene in an adaptive way. It is based on smart sampling of the scene with a small set of masks. These masks are adaptively resized when the part of the scene needs to be recorded with higher resolution. The image is finally recovered by using fast wavelet transforms. Similar ideas have been proposed to improve the performance of ghost and dual photography imaging systems^19^,^20^,^21^. This method is very suitable when the user wants to capture big size images for two main reasons. One, even though the size of the final image can be big (in our experiments, 2048 × 2048 pixels), the number of projected masks remains small due to the nature of the adaptive algorithm. And two, even if this number cannot be reduced due to object characteristics (i.e. objects with very complex spatial features or texture-like images), with this approach only a low resolution set of masks needs to be stored. This characteristic is very suitable from the computational point of view, as small deterministic matrices require low amounts of memory to be stored and can be easily used in fast mathematical operations, providing massive computational gains^22^,^23^. For example, in the recovery stage of the aforementioned image classical SPI techniques would have to store 2048^2^ different masks with 2048^2^ elements each. Nevertheless, with the adaptive approach we are able to recover the image operating with a set of 128^2^ masks with 128^2^ elements each. With these characteristics in mind, we have been able to design a high-resolution fast-operation SPI system with an off-the-shelf DMD and a mid-range laptop. This kind of system can be easily coupled with available commercial microscopes to take advantage from the SPI benefits discussed above.

## Results

### Adaptive Compressive Imaging (ACI)

**Figure 1:**
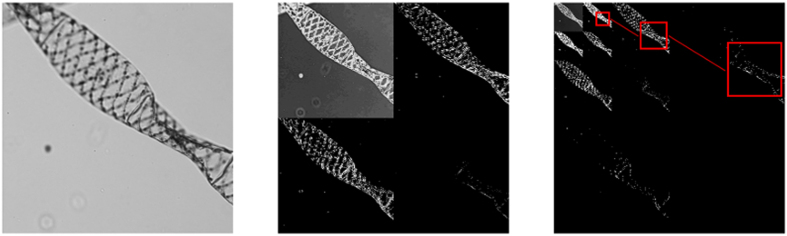
Spirogyra algae image (512 × 512 pixels), its first level and third level wavelet transforms, respectively. The bright pixels on the wavelet transform represent the edges of the scene. In the wavelet representation, a region of the scene is represented by a set of wavelet coefficients arranged in a tree structure, as shown in the right panel.

To better understand the ACI algorithm one has to figure out how the 2D wavelet transform technique works. The process is depicted in Fig. 1. Given an *N* × *N* image, the wavelet transform consists of applying four bandpass filters to the image. As a result, four *N*/2 × *N*/2 quadrants are obtained; a low resolution version of the image and three more quadrants with the information of the horizontal, vertical and diagonal edges. This process can be applied again to the low resolution version of the image, giving the tree-structured image shown in the right image of Fig. 1 where the upper left quadrant (the low resolution image) has been replaced by its wavelet transform. This procedure can be repeated up to 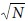 times, when the pixel in the upper left corner contains the total energy of the scene and the rest of the image has the information about the edges of the scene. As it can be seen, the number of pixels containing information of sharp edges is scarce thus few coefficients are enough to get an image similar to the original one. Wavelet compression algorithms choose a number of iterations (levels) and only store the coefficients with values higher than a predetermined threshold, reducing in this way the size of the digital file without a significant loss in quality. As the operations needed to calculate this transform are linear, they are an excellent option for fast algorithms because they require low computational power and memory usage.

**Figure 2:**
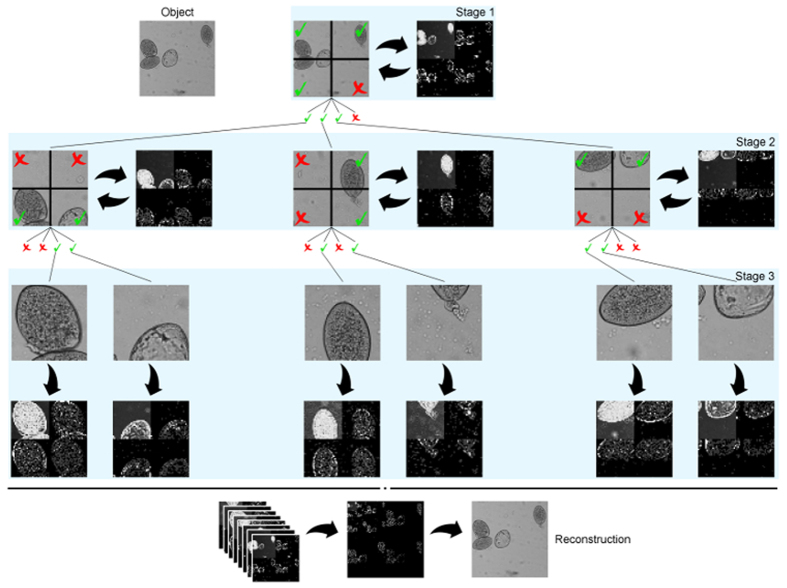
Adaptive Compressive Imaging operation scheme. Object: 256 × 256 *Fasciola hepatica* cells image captured with a commercial microscope. Stage 1: Coarse picture and its level one wavelet transform inspection. As the fourth quadrant has no relevant information, it is discarded. Stage 2: Higher resolution images of the non-discarded zones with their level one wavelet transforms inspection. In this stage, six regions are discarded. Stage 3: Highest resolution images of the non-discarded zones and their level one wavelet transforms. As this is the last stage, no more zones will be discarded so there is no wavelet inspection process. Using all the level one wavelet transforms, the algorithm builds the level three wavelet transform. By doing its inverse wavelet transform, the reconstruction of the scene is acquired. In this example, the total number of measurements to recover the scene was 62% of the 256^2^ measurements established by the Nyquist criterion.

The idea behind the ACI technique is to reduce the number of masks needed to reconstruct an image, using high resolution masks only on the high resolution regions of the image. To that end, we first sample the scene with a low resolution set of masks and only continue sampling with higher resolution masks the parts of the image with regions of interest (i.e. the regions with high density of sharp edges). If the algorithm detects a zone in the scene with no borders, it does not project patterns again on this region, as more resolution is not needed. This process is repeated until we arrive at the final desired resolution. As a demonstration of the technique, in Fig. 2 we show a simulation done with a real microscopy image.

In this case, the goal is to recover an image of 256 × 256 pixels of a group of cells. The set of masks chosen is the 2D Hadamard basis of 64 × 64 pixels. In the first stage, the algorithm acquires a coarse picture of the full scene with our set of masks (resizing the 64 × 64 Hadamard patterns to 256 × 256 pixels). Once the image is recovered, it calculates its level one wavelet transform and searches for the quadrants with higher density of borders. If one of the quadrants has a number of borders below a predefined threshold, it will be discarded on the next stages, thus reducing the total number of masks projected. Once this step is complete, the second stage of the algorithm starts. Now, the 64 × 64 patterns are resized to 128 × 128 pixels, therefore occupying a quarter of the original scene. If none of the quadrants has been discarded in the previous stage, here the algorithm takes four more pictures, recovering then the scene with finer details. If one or more of the quadrants were discarded on the previous stage, the algorithm does not project the set of patterns in the discarded quadrants. In this stage, the algorithm repeats the level one wavelet transformations to each image in order to search again for new zones with no borders. As the algorithm goes on, the search zones get smaller and smaller, and the following sets of smaller masks are only projected on the high spatial resolution zones of the scene. In the last stage, masks are projected onto the regions with finer details of the scene. Once all the regions have been measured with the required resolutions, all the wavelet transforms are used to build the third level wavelet transform shown in Fig. 2. The final 256 × 256 image is recovered via inverse wavelet transform. In contrast with traditional CS techniques, which usually require off-line reconstruction, this step is not computationally consuming. As it will be further discussed in the text, when using ACI this process can be done on the fly with low end computers.

**Figure 3:**
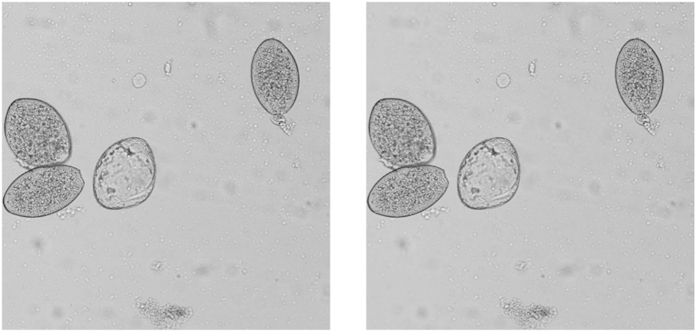
Fasciola hepatica 4 MP image (left panel) and its ACI reconstruction (right panel). The ACI reconstruction is acquired with roughly a 25% of the 2048^2^ measurements stablished by the Nyquist ratio.

The ACI approach has several remarkable benefits when compared with the traditional SPI-CS approach. These can be grouped into three categories: image size, resolution and temporal benefits. In order to prove those benefits, throughout this paper, we will compare our technique with the GPSR-Basic algorithm by Figuereido *et al.*^24^. First, we will discuss the image size benefits. As stated before, to recover a *N* × *N* picture, SPI needs to project *M* = *N*^2^ masks. When using CS techniques, those *M* projections are reduced (typical compression ratios tend to be between 10% and 40% without a significant quality loss). However, this reduction entails the use of convex optimization algorithms to recover the image. The memory and time requirements of the algorithms increase with the size of the image and the number of measurements made. Even if speed is not a crucial requirement for some applications, the memory requirements limit the maximum size of the images recovered. In our experiments, carried on a computer with 24GB of RAM and a Intel Xeon Processor X5690 at 3.47 GHz, the maximum image size that can be reconstructed due to memory limitations with CS algorithms is 256 × 256 pixels, with compression ratios around 50%. When using the ACI algorithm, only a small set of masks of low resolution and the measurements vector need to be stored. In practice, we did simulations of images with sizes up to 4 Megapixel (MP), even though this is not the limit of our equipment. As can be seen in Fig. 3, the regions of interest of the scene are recovered with perfect resemblance, and the zones with no information have lower detail.

When dealing with higher resolutions like the one shown above, the reconstruction time starts to be a crucial factor to be reckoned. A general approach made by researchers is to sacrifice some quality in their reconstructions in order to achieve higher frame rates. This can be made by applying high compression rates or by novel approaches like the one proposed by Radwell *et al.*^18^. This procedures either aren't fast enough to achieve high resolution real time imaging or need a priori information about the scene to speed-up the reconstruction process. In SPI systems, the image acquisition time, *t*_*a*_, depends on the number of projected masks, *M*, which is determined by the size of the image. Defining the projection rate of the SLM as *R*_*SLM*_, the image acquisition time is given by 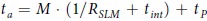, where *t*_*P*_ is the post-processing time to recover the picture from the measurements made, *M*, and *t*_*int*_ is the integration time of the bucket detector. Ideally, both *M* an *t*_*p*_ should be as low as possible. As single-pixel detectors work at higher frequencies than SLM,s, *t*_*int*_ is negligible in all the scenarios considered here where lighting conditions are not extreme. Whereas traditional single pixel imaging requires *M* to be equal to the number of pixels of the image, *N*^2^, and has negligible *t*_*p*_, CS techniques reduce *M* but increase *t*_*p*_. Furthermore, CS techniques need to solve a convex optimization problem to recover an image, which requires high amounts of memory. Adaptive imaging techniques are known to reduce *M* while keeping negligible *t*_*p*_^19^,^20^. Nevertheless, due to the nature of ACI, increasing the size of the scene does not necessarily imply increasing memory requirements. If there is a memory limitation, the number of stages to reconstruct a scene will be increased, and consequently the size of the masks reduced.

**Figure 4:**
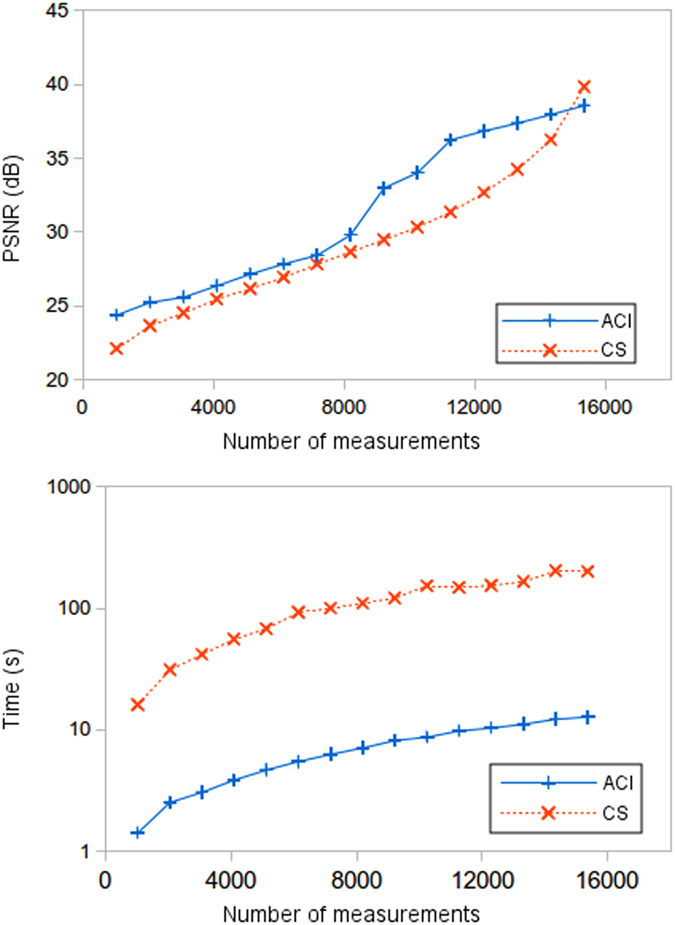
Full frame quality and time comparison between CS and ACI techniques. The full scene resemblance is measured with the PSNR comparison in the top graph. Given the same amount of measurements, the quality is similar in both methods. However, the time comparison shows that the ACI technique outspeeds the traditional CS technique.

**Figure 5:**
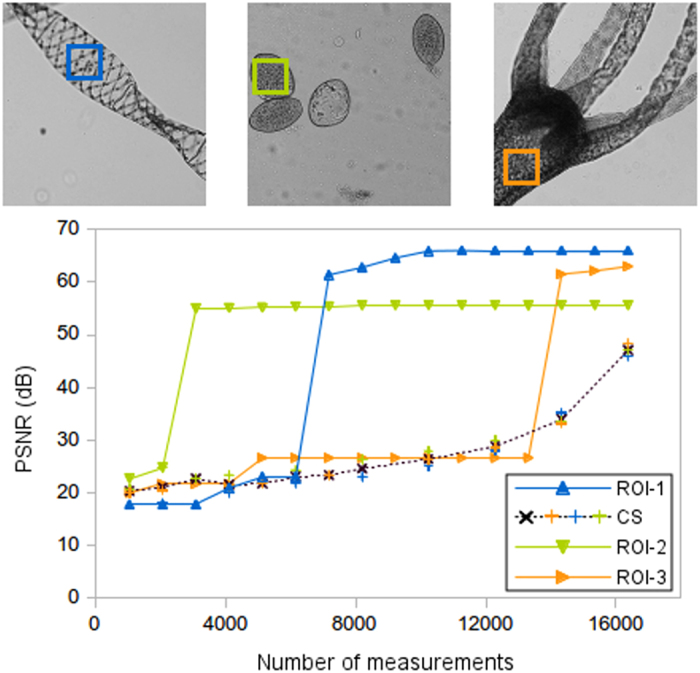
ROI quality comparison between CS and ACI techniques. Three biological samples are selected for the analysis. In each sample, one ROI is studied. Due to the adaptive nature of the ACI approach, its PSNR curves present a steplike behaviour, acquiring higher resemblance than the traditional CS technique. In order to ease the visualization, the CS curve is the average of the three ROI's, as the results were almost equal in all the images.

In Fig. 4 we show two comparisons between CS and ACI algorithms. We have verified the PSNR and reconstruction time to behave similar for several biological images. To carry out the simulation we have used three different biological test images (shown in Fig. 5), with a size of 128 × 128 pixels. The ACI algorithm number of stages was set to three, thus using the 32 × 32 Hadamard masks. The first graph shows the average of the PSNR versus number of measurements for ACI and CS algorithms when the reconstructions are compared with the original images. In the second graph we show a time comparison between both methods. The time includes not only the CS and ACI algorithm computational time but also the masks projecting time. Even though the quality is similar, it is clear by watching at the lower graph, that the ACI technique has a great benefit in reconstruction times. As can be deduced from the results, high quality pictures can be achieved with sub-Nyquist measurement rates around 50%. With those measuerement ratios and state-of-the-art DMD,s, images of 128 × 128 pixels can be acquired at frame rates around 3 Hz (~8000 measurements at 22.7 kHz). Similar tests were made with higher resolutions with results even more favorable to the ACI technique. Images with a resolution of 256 × 256 pixels are reconstructed in less than a minute with the ACI algorithm in comparison with several days with the GPSR routine. Higher resolution images could not be compared as our equipment is not able to reconstruct images with resolutions above 256 × 256 pixels using CS.

Another huge benefit of the technique is that it recovers the regions of interest at high resolution using sub-Nyquist measurement rates. In Fig. 5 we show an example with three biological samples. For each sample, we chose a region of interest and we compare the PSNR of that region when using both techniques. As CS projects masks covering the entire scene, the quality of the whole image gradually improves with the number of projected masks, independently of the region of interest chosen. However, this does not happen with the ACI algorithm because masks are sent to different regions of the scene. In this case, the ACI curves have a steplike behaviour, where each step corresponds to a stage of the algorithm. If there are few regions of interest in the scene, steps are concentrated in the initial measurements (see green curve), while if the scene is plenty of sharp edges, the highest quality of the region is achieved later (see orange curve). In microscopy setups, where the samples usually lay onto specific regions of a slide, this characteristic can be used to recover specimens with very low number of measurements or to locate regions of interest in a fast way. Once those regions are located, the amplification of the system can be changed so the sample fills the full field of view of the system.

### Experimental results

**Figure 6:**
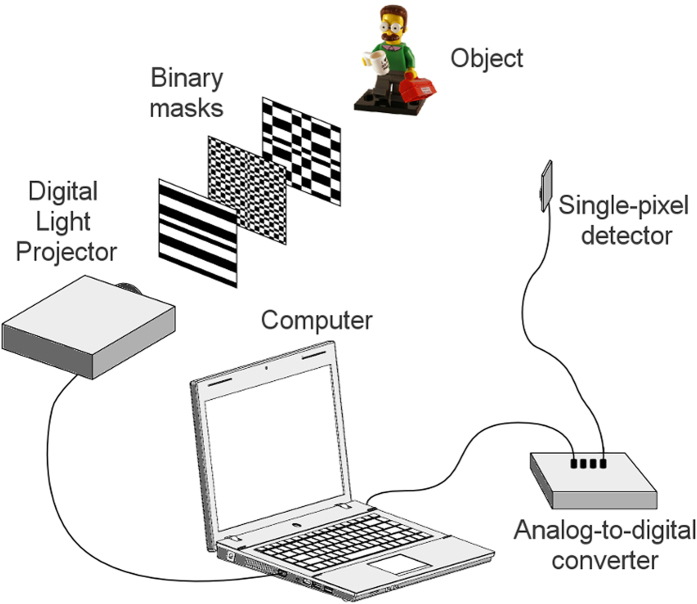
Experimental setup used for ACI reconstructions. The digital projector sends a predefined set of masks to different parts of the object. The light reflected from the object is measured with a bucket detector. The signal is digitalized and used to recover an image of the scene.

In order to test those ideas, we conducted a proof of concept experiment with a projector and a mid-range laptop. The experimental setup is shown in Fig. 6. It consists of a digital light projector, a photodiode, an analog-to-digital converter and a computer. The DLP sends the set of masks onto different regions of the object, resizing them when needed. Each set of resized masks is precomputed, and custom software written in Labview chooses the suitable one for each stage of the algorithm. As the number of pixels of each mask remains the same in all stages (the only change is the pixel size), the reconstruction algorithm computational charge is alleviated. By means of an optical collecting system, light reflected by the object is measured with the photodiode. Being the quantum efficiency of photodetectors higher than CCD/CMOS sensors and given that more photons reach the detector at each measurement, signals acquired suffer less distortion from dark and read-out noise^1^. The analog-to-digital converter digitalizes the signal, and the computer reconstructs the images with custom code written in Matlab. The experimental process of projecting the patterns and measuring the electrical signal is controlled by custom software written in Labview.

**Figure 7:**
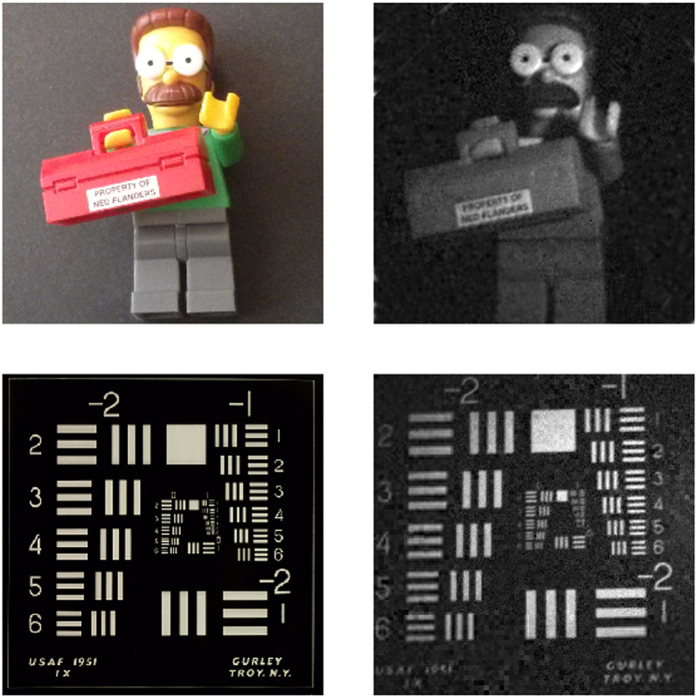
ACI experimental results. In the upper row, we show a 256 × 256 pixels LEGO‚ Ned Flanders picture (left) and its reconstruction via the ACI technique (right). The ACI reconstruction was acquired by using 88% of measurements of the total number of pixels. In the lower row, we show a 512 × 512 pixels USAF1951 test (left) and its ACI reconstruction (right). In this case, the reconstruction was acquired with a 55% of measurements of the total number of pixels. The only post-processing made to the ACI images was white balance correction.

In Fig. 7, two experimental reconstructions are shown. The first scene reconstructed is a small LEGO‚ object. We start the ACI algorithm with 64 × 64 Hadamard patterns and the number of stages is set to 3. Then, the final resolution achieved is 256 × 256 pixels. Unlike standard CS techniques the time needed to reconstruct the scene only depends on the SLM refresh rate and not on the compressive strategy. For this particular reconstruction we have sent 88% of the total 256^2^ measurements established by the Nyquist criterion. In our experiments, we use a DLP LightCrafter 4500 from Texas Instruments. Even though the maximum refresh rate of this device is 4225 Hz, when the number of patterns to be projected gets higher, memory limitations arise. Then, the patterns have to be sent using the video input of the device instead of being preloaded on the internal memory. The speed of this video input is limited to 120 Hz. By encoding 24 different binary patterns in each video frame as a 24-bit image, the maximum speed acquired is 2880 Hz^25^. Bearing that in mind, the acquisition time with our equipment was 20.02 seconds. If state of the art SLM,s are used, with refresh rates around 22.7 kHz and high capacity internal memory, these limitations can be avoided, and reconstruction times of 2.54 seconds can be attained with this method.

As stated before, ACI stands out when capturing big resolution images. Due to the DLP specifications, the biggest square masks that can be projected have a size of 512 × 512 pixels. In the second row of Fig. 7, we show a reconstruction of an USAF test with that resolution. In order to achieve this resolution, four stages of the ACI algorithm were used. As the picture gets bigger, the number of discarded regions tends to get higher, so greater compression ratios are achieved while maintaining good resemblance with the scene. In particular, for this second example we only used 55% of measurements of the 512^2^ stablished by the Nyquist criterion. With our setup, we acquired the image in 50.06 seconds. By using high-end DMD,s, acquisition times of 6.35 seconds could be achieved.

## Discussion

We have designed an ACI algorithm that allows recovering high resolution images at low time costs by using SPI. Compared to traditional CS approaches, we acquire images with equivalent quality in much lower times^1^,^3^. In fact, with the same number of measurements, CS needs post-processing to recover the image while ACI can do it live. Novel approaches like multi-diode design cameras or adaptive ghost imaging have also tackled the speed-resolution limitation with success^20^,^26^. However, our approach maintains the use of a single photodetector, which improves the SNR of the measurements when working on low-light level scenarios, such as biological environments. Furthermore, by using deterministic matrices as the basis of our masks, the method is better suited for fast mathematical operations or even using CS in each stage of the acquisition process^22^. We have also computationally demonstrated the effectiveness of the technique to perfectly recover the regions of interest of an image at sub-Nyquist measurement rates. For the scenes investigated here, containing relatively few regions of intereset, ACI has been shown to provide higher quality reconstructions in less time than the GPSR-Basic CS approach. Compared to other single-pixel techniques, we do not need a priory knowledge of the scene to achieve this speed^18^.

Huge technological efforts are focused on increasing resolution of optical devices. Up to now, SPI has failed to provide high resolution images due to time restrictions. The only limiting factor of ACI technique lies in the SLM refresh rate, enabling us to present high resolution experimental images with dimensions comparable to the SLM number of pixels, without needing stitching techniques and using a single photodiode as a detector.

It has to be also noted that ACI is a very flexible technique. For example, we can use traditional compressive techniques in each ACI stage to reduce even more the number of measurements needed. ACI can also be used to improve the performance of single-pixel cameras working in different regions of the visible, infrared and terahertz spectrum^3^,^4^,^11^. Future work in the ACI technique should be directed towards improving the adaptive scheme. This will involve searching edges in a more intelligent way. Instead of inspecting quadrants regularly placed in the scene, we could freely situate them on high density border zones to gather the information more efficiently. This will improve the quality of the recovered images at even lower measurement rates.

## Methods

The DLP used in the experiments is a DLP LightCrafter 4500 from Texas Instruments. It contains a DMD and three coloured light sources (red, green and blue). A built-in optical system is used to project the patterns onto the scene. The photodetector used is PDA36A-EC from Thorlabs, and the electrical signal is digitalized with NI USB-6001 DAQ. All the experimental results were acquired with a Lenovo ThinkPad E531 laptop with 12GB of RAM and an Intel Core i7 2.20 GHz processor. The biological images used in the simulations correspond to different samples from two slide sets from Carolina (#292148A and #293708).

### ACI algorithm

Here we attach the pseudo-code of the ACI algorithm used in the experimental setup.


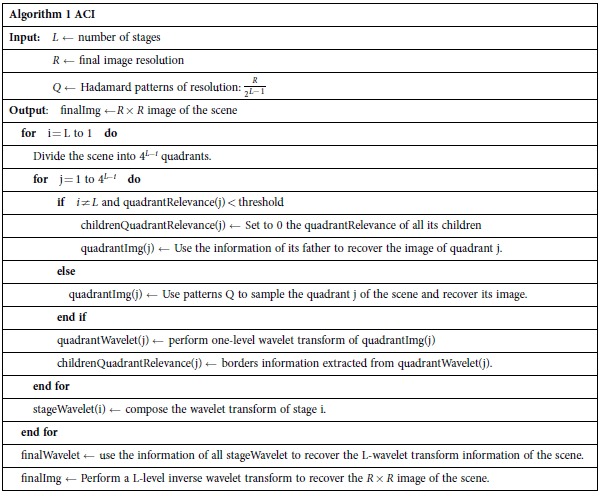


## Additional Information

**How to cite this article**: Soldevila, F. *et al.* High-resolution adaptive imaging with a single photodiode. *Sci. Rep.* **5**, 14300; doi: 10.1038/srep14300 (2015).

## Figures and Tables

**Table 1:** 

**Algorithm 1 ACI**
**Input:** *L* ← number of stages
*R* ← final image resolution
*Q* ← Hadamard patterns of resolution: 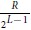
**Output**: finalImg ←*R* × *R* image of the scene
**for** i = L to 1 **do**
Divide the scene into 4^*L*−*i*^ quadrants.
**for** j = 1 to 4^*L*−*i*^ **do**
**if** *i* ≠ *L* and quadrantRelevance(j) < threshold
childrenQuadrantRelevance(j) ← Set to 0 the quadrantRelevance of all its children
quadrantImg(j) ← Use the information of its father to recover the image of quadrant j.
**else**
quadrantImg(j) ← Use patterns Q to sample the quadrant j of the scene and recover its image.
**end if**
quadrantWavelet(j) ← perform one-level wavelet transform of quadrantImg(j)
childrenQuadrantRelevance(j) ← borders information extracted from quadrantWavelet(j).
**end for**
stageWavelet(i) ← compose the wavelet transform of stage i.
**end for**
finalWavelet ← use the information of all stageWavelet to recover the L-wavelet transform information of the scene.
finalImg ← Perform a L-level inverse wavelet transform to recover the *R* × *R* image of the scene.
